# The Treatment of the Local Effects of Cold

**Published:** 1864-02

**Authors:** 

**Affiliations:** Krubieszon.


Art. V.—*The Treatment of the Local Effects of Cold.* By Dr. Krajewski, of Krubieszon.

Cold water and ice play an important part in the treatment of the local injuries induced by intense cold. Whether in the case of mere chilblains in the first or second degree, or real congelation with a livid and cadaverous hue of the affected parts, it is always advisable to begin by frictions with snow, ice or iced-water. The cold application should be continued for several days, and changed twice in twenty hours. In cases in which the cold water appears to be insufficient in itself, its application, perseveringly continued, seems to increase the remedial power of other means which experience points out as the most efficacious.

Among these means, Dr. Krajewski especially recommends the following:

1. Rust's mixture, composed of equal quantities of nitric acid and cinnamon water. The parts affected are painted over with this mixture, an application to be renewed every evening, care being taken to replace it in the day by cold water dressings. This system generally affects a cure of chilblains in the space of twelve days or a fortnight.

2. Jacobi's mixture, composed of equal parts of camphorated brandy and tincture of saffron. This mixture was in great repute in 1812, after the disasters of the Russian campaign. The parts covered with chilblains should be washed several times a day, or be covered with compresses saturated in this fluid.

3. The use of concentrated solution of nitrate of silver (8 gr. for 2½ dr. water), which is strongly recommended by Italian physicians, yielded in the hands of Dr. Krajewski instances of frequent success.

4. Dr. Fitz-Patrick's mixture, consisting of 2½ dr. tincture of iodine and of ½ oz. of soap liniment, applied every evening to the injured parts, after previous lotions in cold water, is another very efficient remedy for chilblains.

5. Glycerine, recently recommended by a physician of Odessa, afforded Dr. Krajewski excellent results, even for ulcerations of parts frozen.

6. Collodion, spread daily over the affected parts, also appears among the remedies from which good effects may be expected in chilblains.

Notwithstanding the use of the different substances we have mentioned, Dr. Krajewski lays much stress on the importance of superintending the general condition of the system. The bad constitution of the patients too often renders the different degrees of chilblain most difficult of cure, and may require the internal use of preparations of iron, cinchona, bitters, anti-scorbutics, etc.

In cases of mortification from cold, Dr. Krajewski advises, according to the precepts generally received, waiting for the appearance of the line of demarcation which indicates the separation of the dead from the living parts, before proceeding to amputation. When two limbs have mortified from freezing, this practitioner unhesitatingly prefers simultaneous to successive amputation of both, and in support of his opinion adduces several cases from the practice of Professors Lebrun of Warsaw, and Zablocki and Inoziemcow of Moscow.

Amputation of a limb at a time, and awaiting the more or less complete cicatrization of the first before attempting the second operation, is a practice which exposes the patient to two dangers at once: phlebitis in the first place, and the absorption of the gangrenous matter, which sometimes forms vast deposits in the midst of the mortified part.

Simultaneous amputation of both limbs occasions no greater reaction than the removal of one, and the general condition of the patients necessarily improves more rapidly than if one limb only had been amputated, leaving permanently in the other the source of infection, which increases the difficulty of the recovery of strength, and may even react unfavorably on the healing of the stump.

### CONFEDERATE STATES
### MEDICAL AND SURGICAL JOURNAL,

